# Cardiac rehabilitation in Austria: long term health-related quality of life outcomes

**DOI:** 10.1186/1477-7525-7-99

**Published:** 2009-12-08

**Authors:** Stefan Höfer, Werner Kullich, Ursula Graninger, Manfred Wonisch, Alfred Gaßner, Martin Klicpera, Herbert Laimer, Christiane Marko, Helmut Schwann, Rudolf Müller

**Affiliations:** 1 Medical University Innsbruck, Department of Medical Psychology, Innsbruck, Austria; 2 Ludwig-Boltzmann-Cluster, Institute for Rehabilitation, Saalfelden, Austria; 3 Austrian Pension Insurance Institution, Pensionsversicherungsanstalt, Vienna, Austria; 4 Center for Cardiac Rehabilitation, St. Radegund, Austria; 5 Center for Cardiac Rehabilitation, Großgmain, Austria; 6 Center for Cardiac Rehabilitation, Hochegg, Austria; 7 Center for Cardiac Rehabilitation, Bad Tatzmannsdorf, Austria; 8 Center for Cardiac Rehabilitation, Felbring, Austria; 9 Center for Cardiac Rehabilitation, Saalfelden, Austria

## Abstract

**Background:**

The goal of cardiac rehabilitation programs is not only to prolong life but also to improve physical functioning, symptoms, well-being, and health-related quality of life (HRQL). The aim of this study was to document the long-term effect of a 1-month inpatient cardiac rehabilitation intervention on HRQL in Austria.

**Methods:**

Patients (N = 487, 64.7% male, age 60.9 ± 12.5 SD years) after myocardial infarction, with or without percutaneous interventions, coronary artery bypass grafting or valve surgery underwent inpatient cardiac rehabilitation and were included in this long-term observational study (two years follow-up). HRQL was measured with both the MacNew Heart Disease Quality of Life Instrument [MacNew] and EuroQoL-5D [EQ-5D].

**Results:**

All MacNew scale scores improved significantly (p < 0.001) and exceeded the minimal important difference (0.5 MacNew points) by the end of rehabilitation. Although all MacNew scale scores deteriorated significantly over the two year follow-up period (p < .001), all MacNew scale scores still remained significantly higher than the pre-rehabilitation values. The mean improvement after two years in the MacNew social scale exceeded the minimal important difference while MacNew scale scores greater than the minimal important difference were reported by 40-49% of the patients.

Two years after rehabilitation the mean improvement in the EQ-5D Visual Analogue Scale score was not significant with no significant change in the proportion of patients reporting problems at this time.

**Conclusion:**

These findings provide a first indication that two years following inpatient cardiac rehabilitation in Austria, the long-term improvements in HRQL are statistically significant and clinically relevant for almost 50% of the patients. Future controlled randomized trials comparing different cardiac rehabilitation programs are needed.

## Background

Besides prolonging life, the objectives of cardiac rehabilitation (CR) include the reduction of symptoms and the improvement of physical functioning and general wellbeing [1,2]. These outcomes are typically considered to be patient-reported outcomes (PRO) and have top-tier priority when it comes to assessing quality in cardiovascular care [3]. A recently published meta-analysis showed that 12 out of 12 exercise-based outpatient CR programs improved health-related quality of life (HRQL) but the magnitude of improvement in HRQL with cardiac rehabilitation exceeded that of the controls in only two trials [4].

There is a great variety of CR programs in the different European countries with either inpatient or outpatient (including home-based CR [5]) or both CR programs available for patients [6]. A wide range of patients having undergone different interventions (e.g. percutaneous coronary intervention (PCI) and coronary artery bypass grafting (CABG) or heart valve surgery (HVS)) and presenting various diagnoses (myocardial infarction (MI), angina or heart failure (HF)) are eligible for these programs which makes it difficult to compare PROs. Inpatient as well as outpatient CR programs are provided in Austria [7] and there is evidence from a non-randomized study that both types of CR programs adequately improve the short term (3-month) outcome of HRQL [8]. In addition, short-term studies including PRO and clinical data for the major six Austrian cardiac inpatient rehabilitation centers have documented statistically significant and clinical important improvements in HRQL and reduction of risk factors in an unselected patient group [9].

Although the Austrian legal framework makes it mandatory that the health care systems and their long-term benefits are evaluated from a patient-centered perspective (Gesundheitsqualitätsgesetz BGBL I Nr. 179/2004), there is little or no data available regarding the long-term (>12 months) effects of inpatient CR programs on HRQL [10]. Further, the question about which particular sub-group within the population of eligible patients enjoys the greatest benefits from these programs within a particular timeframe and in accordance with national [11,12] or international guidelines [13] has not been answered. The aim of this study was therefore to document the long-term PRO improvements of the inpatient CR programs available in Austria.

## Methods

Over a period of 8 weeks in 2004, 487 consecutive patients after MI, angina or heart valve disease with or without PCI, CABG or heart valve surgery in six cardiac rehabilitation centers managed by the Austrian Pension Insurance Institution ("Pensionsversicherungsanstalt" or "PVA") were included in this observational study. Patients completed the 4-week inpatient CR program as soon as possible after initial treatment. A detailed description of the CR program has been published and the selected patient group constitutes a representative sample of the participants in the inpatient CR programs available in Austria [9]. The protocol was approved by the institutional review board of the Austrian Pension Insurance Institution.

Baseline data were collected at the beginning (pre rehabilitation, t0) and at the end of the 4 week inpatient CR (post rehabilitation, one month t1) [9]. The two year follow-up was performed as a postal follow-up (t2). The mailed package included a prepaid return envelope with the two questionnaires used at baseline, the MacNew Heart Disease Health-related Quality of Life Instrument [MacNew] and the EuroQol 5D [EQ-5D] plus a list of major adverse cardiac events. One postal reminder was sent out if patients did not return the initial questionnaire.

### MacNew

The MacNew is an internationally well documented valid and reliable instrument to assess HRQL for patients with different manifestations of heart disease, such as angina pectoris [14], myocardial infarction [15], heart failure [16], and arrhythmia [17] as well as different interventions (such as PCI, CABG [18], pacemaker implant [19] or CR [5,8,20]). Currently the MacNew is the only international disease-specific HRQL instrument that ensures a reliable and valid assessment and comparison of cardiovascular patients with varying presentations and symptoms of their disease.

The MacNew comprises 27 items which are scored from 1 (poor HRQL) to 7 (high HRQL) and consists of three scales: physical limitations, emotional function, and social function; additionally an overall HRQL score can be calculated [21]. Reference data are available for different diagnostic entities and age groups [22]. The minimal important difference (MID; knowledge of the smallest change in instrument score that patients perceive as important [23]) for a MacNew change score has been established to be 0.5 MacNew points [22].

### EQ-5D

The EQ-5D is a generic instrument for the measurement of HRQL and therefore particularly suited for comparisons with other diseases (e.g. cancer). On the basis of the utility approach, the EQ-5D can be used to calculate quality adjusted life years (QALYs) [24]. The EQ-5D consists of a 5-dimensional descriptive system and a visual analogue scale allowing assessment of relevant segments of HRQL: mobility, self-care, usual activities, pain/discomfort, and anxiety/depression. The EQ-5D has repeatedly been used and was validated with the aid of the MacNew in German-speaking CHD patients with acceptable psychometric properties (test-retest reliability and responsiveness) [25].

### Major adverse cardiac events

The patients were queried whether and when the following major adverse cardiac events had occurred in the last two years: 1) heart attack, 2) symptoms of angina, 3) bypass surgery, 4) valve replacement, and 5) coronary intervention. In addition, the patients were asked if they had participated in other rehabilitation interventions such as another inpatient rehabilitation program or a follow-up outpatient rehabilitation program. Death as a major adverse cardiac event was recorded with the help of the Austrian health information system.

### Statistical analysis

Descriptive procedures (means, standard deviation, frequencies) were used to describe patient characteristics. To compare responders with non-responders independent t-test and chi-square were used. Paired t-test (MacNew) and Wilcoxon (EQ-5D) test were applied to check the statistical significance for time and analysis of variance for group comparisons.

Effect sizes for the comparison baseline/follow-up were calculated (ES = (M1-M2)/SD1). Values between 0.20 and < 0.50 are considered as small, values between 0.50 and < 0.80 as moderate and ≥ 0.80 as high [26]. The significance level was established at p < 0.05. All analyses were conducted using the statistics software package SPSS 16 for Windows (SPSS Incorp., USA).

## Results

Questionnaires were returned by 351 patients (mean age of 60.9 ± 12.5 years, 66% males, completion rate of 72.1%, Table 1). Additional selected baseline socio-demographic and clinical variables for all responders and non-responders are given in Table 1. Reasons for not returning the questionnaires included death (14.7%), incorrect address (5.2%), and unknown (80.1%). Compared to responders, non-responders were 2.5 times more likely to have been working at baseline (Table 1). Based on the other available variables no significant difference between responders and non-responders could be detected.

**Table 1:** Clinical- and socio-demographic patient characteristics of responders and non-responders (N = 487)

		Responder			Non-responder			
Variable	Categories	M ± SD	N	%	M ± SD	N	%	p-value
**Age**		60.9 ± 12.5			59.2 ± 13.5			0.08^$^
								
**Gender**	Male		232	66.1%		95	70.8%	
	Female		119	33.9%		41	29.2%	
	Total		351	100%		136	100%	0.33^§^
								
**Professional status**	Employed		43	12.3%		42	30.9%	
	Retired		260	74.1%		63	46.3%	
	Unemployed		9	2.6%		4	2.9%	
	Other		25	7.1%		10	7.4%	
	Total		337	96.0%		119	87.5%	
	Missing		14	4.0%		17	12.5%	0.08^§^
								
**Education**	Compulsory education (CE)		101	28.8%		34	25.0%	
	CE+ vocational training		135	38.5%		54	39.7%	
	University degree		89	25.4%		32	23.5%	
	Total		325	92.6%		120	88.2%	
	Missing		26	7.4%		16	11.8%	0.45^§^
								
**Smoking status**	Current		39	11.1%		11	8.1%	
	Ex-smoker		165	47.0%		69	50.7%	
	Never smoker		119	33.9%		34	25%	
	Total		323	92.0%		114	83.8	
	Missing		28	8.0%		22	16.2%	0.34^§^
								
**Primary diagnosis**	Ischemic heart disease		271	77.2%		109	80.1%	
	Heart valve disease		51	14.5%		13	9.6%	
	Other		29	8.3%		14	32.6%	0.30^§^
								
**Primary intervention**	PCI		170	48.4%		63	46.3%	
	CABG		102	29.1%		39	28.7%	
	HVS		54	15.4%		18	13.2%	
	OPT		25	7.1%		16	11.8%	0.77^§^
								
**NYHA^#^**	I		328	93.4%		118	86.8%	
**(at discharge of initial CR)**	II		19	5.4%		16	11.8%	
	III		4	1.1%		2	1.5%	0.05^§^
	IV		-	-				

PCI = percutaneous coronary intervention

CABG = coronary artery bypass grafting

HVS = heart valve surgery

OPT = optimal pharmacological treatment

^# ^New York Heart Association Classification

^$^independent t-test

^§^chi-square

During the two year follow-up, major adverse cardiac events among the 487 patients were recorded on 140 occasions. It included 20 deaths (4.1%) with angina the most frequent event (11.9%, Table 2). A single major adverse cardiac event was recorded for 76 patients while 21 patients reported more than one.

**Table 2:** Major Adverse Cardiac Events in the last two years post cardiac rehabilitation

Major Adverse Cardiac Event	Frequency N	Percentage %
**Death (all-cause mortality)**	**20**	**4.1%**
Myocardial infarction	10	2.1%
Recurring angina	68	11,9%
CABG	8	1.6%
Heart valve surgery	8	1.6%
Coronary intervention (incl. stinting)	26	5.3%

Total	140	28.7%

Multiple major adverse cardiac events in 21 patients

There was a significant short-term improvement in all MacNew HRQL scales over the one-month inpatient CR program (Table 3, and [9]) with fewer patients (p < 0.001) reporting problems on the EQ-5D mobility, daily activities and pain/discomfort sub-scales at the end of inpatient CR (Table 7 in [9]). It is important to note that both responders and non-responders reported the same initial improvement in all MacNew and EQ-5D HRQL scale scores after CR (global: p = .622; physical: p = .948; emotional: p = .377; social: p = .711, mobility; p = .784; self-care: p = .881; daily activities: p = .451; pain: p = .655; anxiety/depression: p = .293).

**Table 3:** Mean change [M; 95% Confidence Interval (95% CI)] scores in MacNew HRQL, effect size statistics [ES; t0-t2] and minimal important difference [MID; t0-t2] over time

		t0-t1			t1-t2			t0-t2	Deterioration - MID [-0.5]		Unchanged no change = MID		Improvement + MID [0.5+]	
MacNew	N	M	95% CI	p-value^$^	M	95% CI	p-value^$^	ES	N	%	N	%	N	%
Global	340	0.7	0.60 - 0.85	<0.001	-0.4	-0.60 - -0.23	<0.001	0.28	86	24.5	99	28.2	151	43.0
Emotional	320	0.6	0.49 - 0.76	<0.001	-0.4	-0.61 - -0.19	<0.001	0.20	80	22.8	94	26.8	139	39.6
Physical	339	0.9	1.0 - 0.70	<0.001	-0.5	-0.72 - - 0.33	<0.001	0.26	102	29.1	83	23.7	150	42.7
Social	338	0.8	0.67 - 0.98	<0.001	-0.3	-0.55 - -0.13	<0.001	0.40	85	24.2	77	21.9	171	48.7

t0: baseline, pre rehabilitation

t1: post rehabilitation, one month

t2: two years follow-up

ES: effect size statistics from t0 to t2

MID: minimal important difference from t0 to t2 [MID = 0.5 MacNew points]

^$^paired t-test

**Figure 1:**
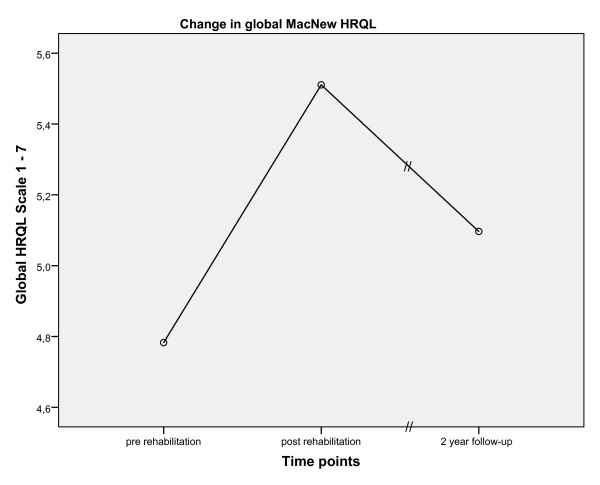
**Change in mean global MacNew HRQL over time**.

Over the two year period following the end of inpatient rehabilitation program, HRQL significantly decreased in all MacNew scales (e.g., global MacNew, Figure 1). However, at the two year follow-up the mean HRQL for all MacNew scale scores were still significantly higher than at baseline with the social HRQL on average still above the MID of 0.5 MacNew points (change in global HRQL: 0.33 p = < .001; physical HRQL: 0.35 p = < .001; emotional HRQL: 0.24 p = .003; social HRQL: 0.52 p = < .001). Although 43.0% [n = 151] of the patients reported an improvement in HRQL over the two-year follow-up that was equal to or exceeded the MID of 0.5 points, the effect sizes were small two years after inpatient CR (Table 3).

On the basis of the MacNew MID, the responders were then grouped as having a negative (-0.5 MacNew points), unchanged [-0.49-0.49 MacNew points) or positive [+0.5 MacNew points] MID (Table 3). About 25% of the patients reported either a clinically important deterioration or remained unchanged with between 40 and 49% of the patients reporting a clinically important improvement on the MacNew HRQL scale scores. Moreover, patients who reported an improved HRQL of greater than the MID two years after CR had initially reported an improvement of 1.12 MacNew points with CR. This initial improvement was significantly higher (p < 0.001) than that reported by either those whose HRQL had deteriorated within two years (initial improvement = 0.38 MacNew points) or those whose HRQL remained unchanged over two years (initial improvement = 0.42 MacNew points).

On the basis of the change in MacNew global HRQL, the effect sizes and the proportion of patients exceeding the MacNew MID of 0.5 points were calculated for the responders in accordance with the main diagnosis, the pre-treatment, the risk profile, and the socio-demographic status (age groups and gender) (Additional file 1; Table S1). HRQL improved at two years in patients with ischaemic heart disease (p < 0.002) with a medium effect size of 0.65 and an improvement greater than the MID in 43.5%; HRQL also improved in patients with heart valve disease (p = 0.011) with a small effect size of 0.42 and an improvement greater than the MID in 49%. As far as the treatment before CR is concerned, HRQL improved over the two years in patients with CABG and HVS (p < 0.001) with medium effect sizes of 0.60 and 0.64, respectively, and an improvement greater than the MID in 58.4% of the patients after CABG and in 60.6% of patients having undergone HVS.

Patients without hypertension, without diabetes or without hypercholesterolemia improved their HRQL (p < 0.001) at two years with a small (no diabetes, ES = 0.38) or medium effect sizes (no hypertension, ES = 0.64; no hypercholesterol, ES = 0.56) and an improvement greater than the MID two years after CR in 48-56% of the patients. Patients with no major adverse cardiac event in the last two years reported on average a medium effect, with 51% having a long lasting effect. In contrast, patients with one or more major adverse cardiac events deteriorated on average by 0.3 MacNew points. It is interesting to note that these patients had already initially (t0) low HRQL scale scores (p < 0.001). Two years after CR, HRQL improved (p < 0.001) in male but not in female patients, although the effect size was rather small (ES = 0.37) despite the fact that 48% of the males reported an improvement greater than the MID.

Compared with mid-age (41-65) and older (>65) patients the young (<41) showed strong effects, that were long lasting in 62% of the cases; although statistically significant effects could be shown for the age groups 41 and older, in terms of effect statistics there were no (age group 41-65) or small effects (age group 65+), with long lasting effects for 43-40% of the patients.

Beyond the often long lasting improvements in disease-specific HRQL, no long-term improvements were observed in "mobility", "self-care" and "pain" as measured by the generic EQ-5D. The ability to perform usual daily life activities remained 6% higher two years after CR. However a considerable proportion of patients (7% increase) reported some problems with "anxiety/depression" two years after CR (Table 4). The overall subjective health status based on the EQ-5D VAS Scale returned to baseline level (65.0).

**Table 4:** EQ-5D generic HRQL at baseline (t0), one month (t1) and two years (t2) (percentage %)

	Timepoint				
	t0	t1	t2	Z	p-value [t0-t2]
EQ-5D Dimensions					
Mobility (N = 291)					
No problems	71.3%	83.2%	72%		
Some problems	28.7%	16.3%	27.4%		
Severe problems	0%	0.5%	0.6%	.630	.529^$^
Self care (N = 293)					
No problems	90.5%	93.7%	87.4%		
Some problems	8.4%	4.5%	10.9%		
Severe problems	1.1%	1.8%	1.8%	1.376	.169^$^
Daily activities (N = 290)					
No problems	58.3%	64.0%	64.3%		
Some problems	32.5%	30.6%	31.1%		
Severe problems	9.2%	5.4%	4.6%	1.999	.046^$^
Pain (N = 291)					
No problems	29.0%	40.8%	32.4%		
Some problems	65.5%	55.2%	60.9%		
Severe problems	5.5%	4.1%	.8%	.865	.387^$^
Anxiety/depression (N = 291)					
No problems	65.9%	68.9%	60.9%		
Some problems	31.0%	28.6%	35.8%		
Severe problems	3.1%	2.6%	3.3%	2.167	.030^$^
VAS					
Median (N = 263)	66.0	75.0	70.0		
Mean (N = 208)	63.7 ± 17.6	73.4 ± 17.6	65.0 ± 20.4		.931^#^

VAS Visual Analogue Scale

# T-test

^$^Wilcoxon-Test

t0: baseline, pre rehabilitation

t1: post rehabilitation, one month

t2: two years follow-up

## Discussion

In this study, although mean HRQL decreased over the two years following CR in the 351 patients referred to the six participating Austrian inpatient rehabilitation centers, all MacNew scale scores HRQL remained significantly higher than at baseline with the mean social HRQL change greater than the MID of 0.5 MacNew points. An additional indication for the positive long-term results of inpatient CR can be seen in the fact that as many as 60.1% of the patients reported an improved global MacNew HRQL score with 43.0% achieving or exceeding the MID. Comparing our results to published norm data, baseline HRQL values were below, t1 HRQL values were higher and t2 HRQL values were comparable to published norm values [22].

More detailed analyses made it possible to identify subgroups of patients who benefit most from the programs offered by the PVA inpatient CR centers in Austria. The improvement in HRQL showed greater effect sizes for patients with ischemic heart disease than for patients with valvular disease. Patients having undergone surgery (either CABG or HVS) prior to rehabilitation benefited more than patients after PCI which may be a consequence of the particular positive effect of the PCI that makes these patients report the greatest improvements in HRQL after a PCI but before rehabilitation [27]. This observation questions the additional benefit of inpatient CR as an opportunity for further improvement of HRQL in this patient group. This is in line with previous findings which have shown that CR is especially beneficial to CABG patients 12 months after CR (effect sizes i.e. 0.66 after 12 months) [10].

The management of patients with risk factors, i.e., smoking, hypertension, diabetes mellitus or hypercholesterolemia, where no long-term effects of statistical or clinical significance were observed, is a more challenging task. This raises the question whether it is possible to better manage patients at risk by providing additional on-going support (i.e. outpatient programs, long-term monitoring via modern media - eHealth) with the aim that this would bring about a potentially long-term benefit for these high risk patients.

The results of this study also suggest that younger patients, with an effect size of 0.91, derive the greatest long-term benefit from inpatient CR in terms of an improved HRQL (with 63% having a long-lasting effect). This is partly in line with previous findings, where increased age (65+) was associated with mental HRQL comparable to community norms [28] or with a greater improvement after CR [10]. Our results, however, indicate that there is a U-form type of relationship. Either patients <41 or patients 65+ years old reported small to medium effect sizes, with no effects for the age group 41-65. Finally, the fact that male patients show a greater benefit than female ones may suggest a possible programmatic gender issue which needs further investigation.

The 5 dimensions of HRQL as measured by the generic EQ-5D did show improvements for daily activities for a small proportion of patients (6%). However, in contrast to the disease-specific significant MacNew HRQL changes, the EQ-5D did not pick up an overall global health status improvement for the whole group, with values returning to pre-rehabilitation levels. This is in line with previous research demonstrating that disease specific instruments are more sensitive to change in contrast to generic HRQL instruments which are more useful when comparing different diagnoses [29]. Another important and more general finding is the reported increase in anxiety/depression two years after the end of the inpatient CR program. Since there is presently a controversial discussion about depression as a potential risk factor or a significant comorbidity [30-32] influencing the outcome [33], special attention needs to be paid to the diagnosis of anxiety/depression in patients attending inpatient CR [34].

Although patients with one or more major adverse cardiac events two years after CR reported the same initial improvement of 0.7 MacNew points as patients without major adverse cardiac events in the follow-up period, their initial HRQL scale scores were significantly lower (<4.5 MacNew points) which means that the difference between the two groups was close to the MID of 0.5 MacNew points. Low HRQL has been shown to have negative effects on adherence [35], and adherence itself is a highly relevant factor for health outcomes (e.g. [36,37]). In addition, a previous study using the MacNew scale scores predicted adverse cardiac events including death [38]. This corroborates the findings of the present study suggesting that initial screening for HRQL, especially at the beginning of CR, may be a potential decision-making tool, to improve the identification of high risk patients. An intensive monitoring of high risk patients is advisable (i.e. with low initial HRQL: <4.5 MacNew points at the beginning of CR; or little HRQL improvement: <0.5 MID improvement) after the end of CR in order to prevent possible future major adverse cardiac events. Future studies and programs should address the benefit of ongoing brief contacts with patients having undergone CR with the aim of monitoring their health status with the help of modern eHealth technologies.

The amount of publications addressing PROs such as HRQL after inpatient cardiac rehabilitation is very limited. Published articles addressing inpatient cardiac rehabilitation primarily focus on cost analysis [39], cardiovascular risk factors [40,41], consumer parameters (such as treatment satisfaction or patient expectations [42,43]) or its utilization [44,45]. In this study we documented the long-term (two years) HRQL outcome of patients following a one month inpatient CR. If HRQL has been used as an outcome parameter in cardiac rehabilitation evaluation studies a variety of measures have been used making outcome comparisons difficult [46]. For example Müller-Nordheim used in a similar study the generic SF-36 with a one year follow-up period. The general findings are comparable to ours, with large effect size HRQL improvements for patients after CABG, less after PCI and non after MI [10]. In contrast to our results, women reported more frequently improvements in health status then men. Overall, a consistent application of a single core heart disease specific HRQL outcome measure to allow program comparisons is warranted.

A major limitation of this study is the lack of a control group, which does not allow attributing the documented improvement in HRQL only to CR. As there is no evidence for the natural history of long-term recovery of patients not attending CR in Austria, it is difficult to distinguish the effect of CR from other factors. Further, although there is relatively little documentation of the long-term benefits of inpatient CR, it is a clinically well-established practice in Austria making the feasibility of randomization to a control versus an inpatient CR group questionable. In relation to this, a study by Benzer et al. comparing inpatient CR, outpatient CR and usual care (non-CR participants) over a short period of time indicated that there is a faster recovery of HRQL for CR attendees in contrast to no CR [8]. However the question of whether and how CR non-participants improve over a long term period such as two years remains unanswered and future evaluation projects need to consider the possibility of control groups at least for short-term outcomes (i.e. waiting-list controls). Further, the HRQL of non-responders (27.9%) remains unclear. It should be noted, however, that the analyses of non-responders and responders did not suggest that there was a selection bias based on the available variables, or the initial HRQL improvements. Another limitation of this study is the lack of information about stroke as an additional major cardiac event.

This study documented improved HRQL for as many as 49% of all patients two years after CR, complementing the available literature on long term health outcomes after inpatient CR. In particular male patients up to 41 years with either ischemic heart disease or pre-treated with CABG or HVS and without risk factors benefited most from the existing CR programs. It may, therefore, be necessary to develop gender- and age-specific modules. HRQL screening for high risk patients (low HRQL) combined with a long term monitoring should be applied to minimize major cardiac events in high risk patients. Future controlled randomized trials comparing different cardiac rehabilitation programs using a single core heart disease specific PRO outcome measure are needed.

## Competing interests

The authors declare that they have no competing interests.

## Authors' contributions

SH drafted the manuscript and performed the statistical analysis. UG, WK and RM designed the study protocol. MW, AG, MK, HL, CM and HS organized and carried out the original study. All authors read and approved the final manuscript.

## Supplementary Material

Additional file 1 **Table S1**. Mean change [M ± standard deviation, SD], effect size statistics [ES; t0-t2] and minimal important difference [MID; t0-t2] over time in global MacNew HRQL scores according to subgroups. Click here for file
