# Supplementary material for: Cardiac rehabilitation in Austria: long term health-related quality of life outcomes
Source: Health Qual Life Outcomes. 2009 Dec 8;7:99. doi: 10.1186/1477-7525-7-99 (PMC3224906; doi:10.1186/1477-7525-7-99)
Supplement: Additional file 1 — Table S1. Mean change [M ± standard deviation, SD], effect size statistics [ES; t0-t2] and minimal important difference [MID; t0-t2] over time in global MacNew HRQL scores according to subgroups. [file 1477-7525-7-99-S1.DOC]

Table S1: Mean change [M ± standard deviation, SD], effect size statistics [ES; t0-t2] and minimal important difference [MID; t0-t2] over time in global MacNew HRQL scores according to subgroups

|  |  |  |  |  |  |  |  | Patients improving | Patients unchanged | Patients deteriorating |
| --- | --- | --- | --- | --- | --- | --- | --- | --- | --- | --- |
|  |  | t0 | t1 | t2 | Δ t0 –t2 | p^#^ | ES^§^ | + MID†  [0.5+] (%) | no change  = MID (%) | - MID  [-0.5] (%) |
| Main diagnosis |  |  |  |  |  |  |  |  |  |  |
|  | Ischemic heart disease (N^1^=260) | 4.88 (±1.1) | 5.52 (±1.0) | 5.14 (±1.3) | 0.27 (±1.4) | **.002** | **0.65** | 43.5% | 30.8% | 28.8% |
|  | Heart valve disease (N^1^=49) | 4.53 (±1.2) | 5.32 (±1.2) | 5.08 (±1.4) | 0.58 (±1.5) | **.011** | **0.42** | 49.0% | 26.5% | 24.5% |
|  | Other (N^1^=27) | 4.87 (±1.1) | 5.83 (±1.0) | 5.30 (±1.3) | 0.47 (±1.4) | .083 | **0.39** | 51.9% | 22.2% | 25.9% |
|  | p-value^$^ | .12 | .17 | .77 | .31 |  |  |  |  |  |
|  |  |  |  |  |  |  |  |  |  |  |
| Pre treatment |  |  |  |  |  |  |  |  |  |  |
|  | PCI (N^1^=155) | 4.91 (±1.1) | 5.47 (±1.1) | 5.03 (±1.3) | 0.09 (±1.4) | .41 | 0.11 | 35.7% | 33.1% | 31.2% |
|  | CABG (N^1^=89) | 4.72 (±1.1) | 5.59 (±0.9) | 5.39 (±1.1) | 0.63 (±1.4) | **<.001** | **0.60** | 58.4% | 25.8% | 15.7% |
|  | HVS (N^1^=33) | 4.44 (±1.3) | 5.34 (±1.2) | 5.28 (±1.2) | 0.97 (±1.5) | **<.001** | **0.64** | 60.6% | 15.2% | 24.2% |
|  | OPT (N^1^=22) | 4.91 (±0.8) | 5.74 (±0.6) | 5.06 (±1.3) | 0.11 (±1.4) | .72 | 0.19 | 40.9% | 27.3% | 31.8% |
|  | p-value^$^ | .04^b^ | .32 | .17 | .001^a.b^ |  |  |  |  |  |
|  |  |  |  |  |  |  |  |  |  |  |
| Risk profile |  |  |  |  |  |  |  |  |  |  |
|  | Hypertension (N^1^=205) | 4.79 (±1.1) | 5.46 (±1.1) | 4.93 (±1.3) | 0.15 (±1.4) | .14 | 0.13 | 38.5% | 31.2% | 30.2% |
|  | No | 4.94 (±1.0) | 5.64 (±1.0) | 5.58 (±1.1) | 0.65 (±1.2) | **<.001** | **0.64** | 56.0% | 26.7% | 17.2% |
|  | Diabetes Mellitus (N^1^=91) | 4.72 (±1.1) | 5.41 (±1.0) | 4.87 (±1.3) | 0.18 (±1.4) | .24 | 0.13 | 37.4% | 31.9% | 30.8% |
|  | No | 4.89 (±1.0) | 5.6 1(±1.0) | 5.27 (±1.2) | 0.38 (±1.4) | **<.001** | **0.38** | 48.2% | 27.2% | 24.6% |
|  | Hypercholesterol (N^1^=210) | 4.88 (±1.1) | 5.53 (±1.1) | 5.06 (±1.2) | 0.19 (±1.4) | .05 | 0.15 | 41.9% | 29.5% | 26.6% |
|  | No | 4.75 (±1.0) | 5.52 (±1.0) | 5.31 (±1.3) | 0.57 (±1.3) | **<.001** | **0.56** | 49.5% | 29.4% | 21.1% |
|  |  |  |  |  |  |  |  |  |  |  |
|  |  |  |  |  |  |  |  |  |  |  |
| MACE^2^ |  |  |  |  |  |  |  |  |  |  |
|  | Yes (N^1^=95) | 4.51(±1.2) | 5.2(±1.2) | 4.2(±1.3) | -0.3 (-+1.6) | .078 | **-0.26** | 28.9% | 27.8% | 43.3% |
|  | No (N^1^=252) | 4.94 (±1.0) | 5.6(±1.0) | 5.5(±1.1) | 0.6 (±1.3) | <.001 | 0.56 | 50.8% | 30.1% | 19.1% |
|  | p-value^$^ | <.001 | <.001 | <.001 | <.001 |  |  |  |  |  |
|  |  |  |  |  |  |  |  |  |  |  |
| Socio-demographic status | Socio-demographic status |  |  |  |  |  |  |  |  |  |
| Gender | Male (N^1^=223) | 4.87 (±1.1) | 5.52 (±1.0) | 5.28 (±1.2) | 0.41 (±1.4) | **<.001** | **0.37** | 48.0% | 28.3% | 23.8% |
|  | Female (N^1^=113) | 4.74 (±1.2) | 5.52 (±1.1) | 4.88 (±1.4) | 0.17 (±1.4) | .22 | 0.12 | 38.9% | 31.9% | 29.2% |
|  | p-value^$^ | .302 | .977 | .006 | .127 |  |  |  |  |  |
|  |  |  |  |  |  |  |  |  |  |  |
| Age groups | 18 – 40 years (N^1^=16) | 4.92 (±0.9) | 5.89 (±0.9) | 5.69 (±1.1) | 0.77(±1.0) | **.009** | **0.91** | 62.5% | 25.0% | 12.5% |
|  | 41 – 65 years (N^1^=163) | 4.82 (±1.1) | 5.45 (±1.2) | 5.02 (±1.4) | 0.23(±1.4) | **.045** | 0.18 | 43.4% | 28.9% | 27.7% |
|  | 65+ years (N^1^=122) | 4.91 (±1.1) | 5.53 (±0.9) | 5.23 (±1.2) | 0.32(±1.5) | **.002** | 0.27 | 40.2% | 32.8% | 27% |
|  | p-value^$^ | .753 | .357 | .091 | .334 |  |  |  |  |  |

^1^N based on complete data for t0-t2

^2^ Major adverse cardiac events

t0: baseline, pre rehabilitation

t1: post rehabilitation, one month

t2: two years follow-up

p^#^ within group changes t0-t2 (paired t-test)

p^$^ between group differences (analysis of variance)

ES^§^ effect size between t0 and t2

MID† minimal important difference

^a^ PCI vs. CABG

^b^ PCI vs. HVS
